# Proton Transfer via Arginine with Suppressed p*K*_a_ Mediates Catalysis by Gentisate and Salicylate Dioxygenase

**DOI:** 10.1021/acs.jpcb.4c03164

**Published:** 2024-07-09

**Authors:** Qian Wang, Aleksey Aleshintsev, Kamal Rai, Eric Jin, Rupal Gupta

**Affiliations:** † Department of Chemistry, College of Staten Island, City University of New York, Staten Island, New York 10314, United States; ‡ Staten Island Technical High School, Staten Island, New York 10306, United States; § Ph.D. Programs in Biochemistry and Chemistry, The Graduate Center of the City University of New York, New York, New York 10016, United States

## Abstract

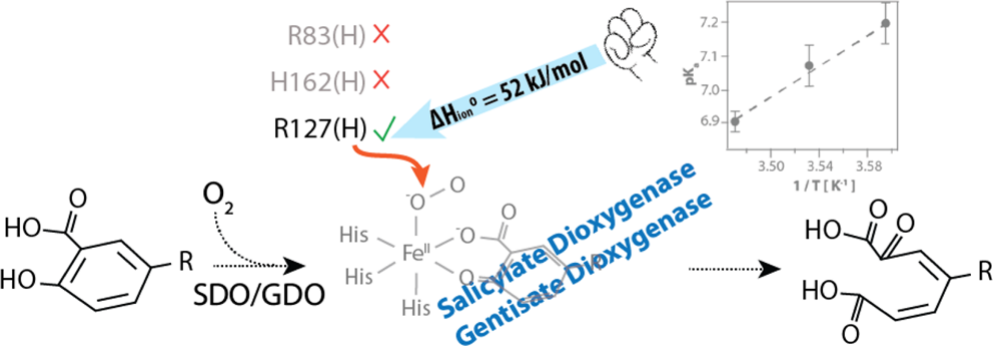

Gentisate and salicylate 1,2-dioxygenases (GDO and SDO) facilitate aerobic degradation of aromatic rings by inserting both atoms of dioxygen into their substrates, thereby participating in global carbon cycling. The role of acid–base catalysts in the reaction cycles of these enzymes is debatable. We present evidence of the participation of a proton shuffler during catalysis by GDO and SDO. The pH dependence of Michaelis–Menten parameters demonstrates that a single proton transfer is mandatory for the catalysis. Measurements at variable temperatures and pHs were used to determine the standard enthalpy of ionization (Δ*H*_ion_°) of 51 kJ/mol for the proton transfer event. Although the observed apparent p*K*_a_ in the range of 6.0–7.0 for substrates of both enzymes is highly suggestive of a histidine residue, Δ*H*_ion_° establishes an arginine residue as the likely proton source, providing phylogenetic relevance for this strictly conserved residue in the GDO family. We propose that the atypical 3-histidine ferrous binding scaffold of GDOs contributes to the suppression of arginine p*K*_a_ and provides support for this argument by employing a 2-histidine-1-carboxylate variant of the enzyme that exhibits elevated p*K*_a_. A reaction mechanism considering the role of the proton source in stabilizing key reaction intermediates is proposed.

## Introduction

Metalloenzymes activate dioxygen to perform energetically and kinetically demanding chemical reactions such as C–H and C–C bond cleavages. Among these catalysts, nonheme iron-containing oxygenases are the most prevalent family of enzymes, performing dioxygen and substrate activation. Nonheme enzymes, such as gentisate 1,2-dioxygenase (GDO), which cleave aromatic rings, play a critical role in the earth’s atmosphere by participating in global carbon recycling.^1^ Exploring the reaction mechanism of these enzymes is of utmost interest given their predominance and the ability to degrade a diverse set of substrates while maintaining chemical specificity. GDO, a cupin dioxygenase, facilitates aerobic degradation of its dihydroxylated substrate, gentisate, by incorporating both oxygen atoms into the aromatic ring.^2−4^

**Scheme 1:**
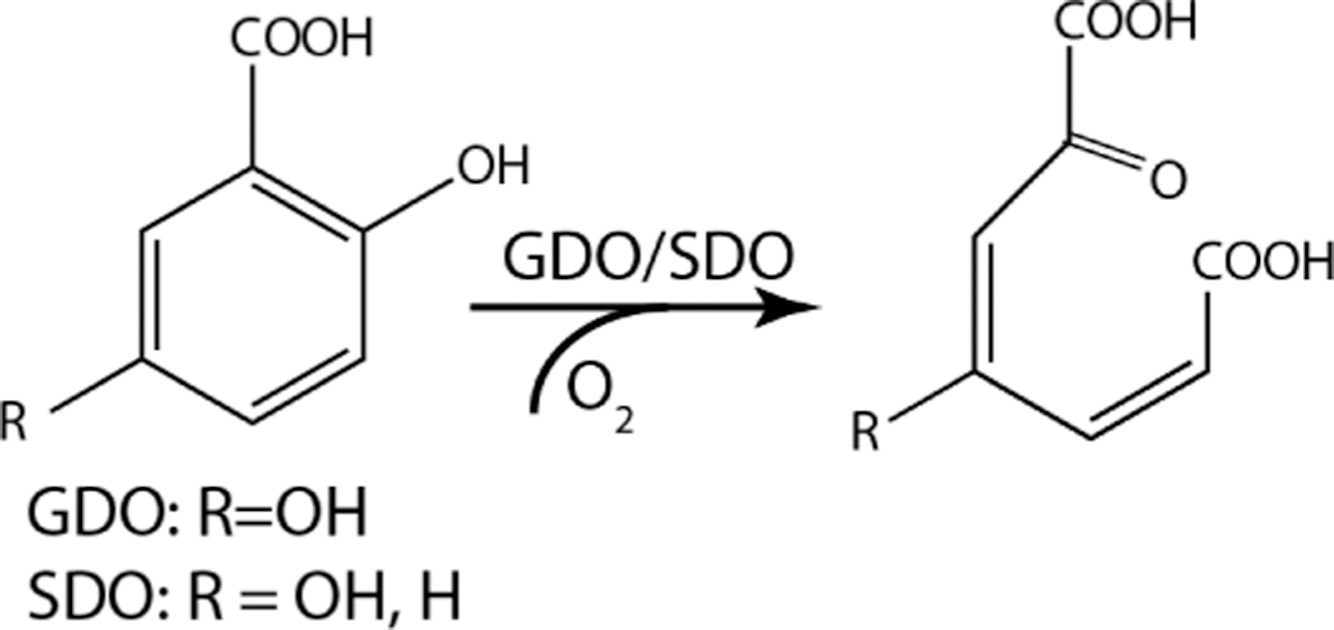
Reaction Catalyzed by GDO and SDO GDO can catalyze gentisate (R = OH), while SDO can degrade both gentisate (R = OH) and salicylate (R = H).

Cupin dioxygenases comprise a large family of enzymes bearing a conserved β-barrel motif. Owing to this motif, these enzymes are structurally distinct from other well-known extra- and intra-diol dioxygenases such as homoprotocatechuate 2,3-dioxygenase (HPCD) and protocatechuate 3,4-dioxygenase (PCD), respectively. Furthermore, members of the cupin dioxygenase family, which is believed to have evolved independently, exhibit remarkable functional diversity by transforming a variety of aromatic substrates.^5^ While significant progress has been made in elucidating the reaction mechanisms of extra- and intra-diol dioxygenases,^6^ the catalytic cycles of cupin dioxygenases (such as GDO), which bear striking features compared to other dioxygenases, largely remain elusive. Enabled by a ferrous cofactor tethered to each monomeric unit, homotetrameric GDOs transform gentisate and its analogues with substitutions at positions 3 and 4 of the aromatic ring. Salicylate 1,2-dioxygnease (SDO) is a unique member of the GDO family, with its unique ability to transform monohydroxylated substrates such as salicylate and several of its substituted analogues (Scheme 1).^7^

Among aromatic ring cleaving dioxygenases, two key features make GDO (and SDO) distinct from their counterparts. First, the Fe cofactor in GDO is tethered to a 3-His binding motif in contrast to the ubiquitous 2-His-1-carboxylate facial triad.^8^ Second, generally, aromatic ring scission at positions 3 and 4 is supported by a ferrous center (such as in extradiol HPCD) and cleavage at positions 1 and 2 is enabled by a ferric cofactor (for instance, in intra-diol PCD). However, the GDO family facilitates ring scission at positions 1 and 2 with the aid of a ferrous cofactor. We note that a homogentisate 1,2-dioxygenase performs ring degradation at positions 1 and 2 with the aid of a ferrous center tethered to a 2-His-1-carboxylate; however, unlike other enzymes mentioned above, including GDO, where the substrate ligates to the Fe center in a bidentate mode, binding of the substrate in homogentisate 1,2-dioxygenase takes place in a monodentate manner. These similarities and differences warrant a detailed evaluation of the reaction mechanisms of these enzymes in the context of their substrate and chemical specificity.

Degradation of the aromatic ring by intra- and extradiol dioxygenases has been extensively investigated.^6^ Reaction intermediates in the catalytic cycles of HPCD and PCD have been spectroscopically, structurally, and computationally characterized.^9−13^ In contrast, no spectroscopic characterization of reaction intermediates of GDO has been conducted. Within the GDO family, SDO is the most structurally characterized member, and several substrate-free and substrate-bound structures of the wildtype enzyme and its variants have been reported in the literature.^14−16^ SDO bears 70% sequence similarity to other members of the GDO family but exhibits remarkable substrate promiscuity.^17^ Based on these observations, proposals in the literature suggest that the reaction mechanism for the catalysis of the dihydroxylated (gentisate) and monohydroxylated (salicylate) substrate may not be identical. *Given the high degree of sequence homology between GDO and SDO, what factors enable the broad range of substrate selectivity while maintaining the chemical selectivity?* Due to the lack of any experimental mechanistic studies, these questions remain unanswered.

Many enzymatic reactions are assisted by acid–base catalysts present in the vicinity of the metal cofactor. For extra- and intra-diol dioxygenases, the proton shufflers have been identified, and their role in catalysis has been established. For instance, a histidine residue (H200) in HPCD has been shown to serve as a proton source stabilizing the superoxo and hydroperoxo intermediates in the catalytic cycle, thereby playing a critical role in the reaction outcome.^9^ Such interactions may also be present in the catalytic cycles of GDO and SDO. Crystal structures of these enzymes show several ionizable amino acids, which may act as a proton source during the reaction. In the absence of experimental studies, quantum mechanical and molecular mechanics (QM/MM) studies have explored the reaction mechanism of SDO.^18−20^ However, these reports offer conflicting views on the role of a proton shuffler in the reaction cycle (vide infra).

In this report, we provide experimental evidence for the presence of a single proton source in the reaction cycles of GDO and SDO. pH dependence of steady-state turnover parameters demonstrates that a proton source with apparent p*K*_a_ between 6.0 and 7.0 is needed for the degradation of gentisate (by GDO and SDO) and salicylate (by SDO). Based on the crystal structures of substrate-bound SDO His162, Arg83 or Arg127 residue could be responsible for proton shuffling. Temperature and pH dependence of Michaelis–Menten parameters allow for the measurement of enthalpy of ionization and suggest that the observed proton source is an arginine residue. These results show that the catalytic cavity suppresses the apparent p*K*_a_ of arginine, reducing its magnitude to that of the histidine residue in an HPCD reaction cycle. We propose that the 3-His motif contributes to the depressed p*K*_a_ of the arginine residue and provides evidence by employing a 2-His-1-carboxylate variant of the enzyme. These findings also provide the phylogenetic relevance of the strictly conserved arginine residue in the GDO family of enzymes. Lastly, our results indicate that the putative superoxo and hydoperoxo intermediates in the catalytic cycle may be stabilized by the proton source and provide insights into the catalytic cycle of GDOs.

## Materials and Methods

### Sample Preparation

SDO and GDO were expressed in BL21(DE3) *Escherichia coli* strain and purified as described in our previous work.^21^ Briefly, both enzymes were overexpressed via plasmids containing their cDNA sequences (SDO from *Pseudaminobacter salicylatoxidans* and GDO from *Corynebacterium glutamicum*) cloned into a pET-41a+ plasmid leaving a His-tag and a tobacco etch virus (TEV) protease cleavage site at the N-terminal of the open reading frame. Cell pellets suspended in a Tris 50 mM, sodium chloride 300 mM, and imidazole 5 mM (pH 8.0) buffer were sonicated, and the enzymes of interest were isolated using a HisTrap column. The purity and yield were established using SDS-PAGE gel and absorbance at 280 nm assuming a molar extinction coefficient of 74.0 mM^–1^ cm^–1^. The His-tag was catalytically removed with TEV protease. Previously established spectrophotometric protocols were then used to estimate the iron occupancy of purified enzymes, which varied in the range of 70–80% between different batches.^22,23^ Briefly, ∼100 μM protein was acid-hydrolyzed with concentrated sulfuric acid and denatured by heat treatment at 95 °C for 15 min. The resulting supernatant, treated with 3 M acetate buffer, 1% NH_2_OH, and 0.3% bathophenanthrolinedisulfonic acid to achieve a resulting pH of 4.5, was incubated at room temperature for 30 min. Fe quantitation of this solution was performed spectrophotometrically by monitoring the absorbance at 535 nm (ε = 22.1 mM^–1^ cm^–1^). The purified enzymes were subsequently concentrated to a final concentration of 1 mM. 100 mM stock solutions of each substrate (gentisate and salicylate) were prepared at neutral pH for subsequent experiments.

### Steady-State Kinetic Measurements

Both enzymes were dissolved in a mixed buffer solution containing MES (20 mM), HEPES (20 mM), CHES (20 mM), and sodium chloride (100 mM), covering a pH range from 5.5 to 9.5. The final working concentrations of GDO and SDO were adjusted to 50 nM (0.5 mg) and 100 nM (1 mg), respectively, to achieve steady-state conditions. The substrate concentrations during steady-state kinetic experiments were varied between 50 and 4000 μM. The Michaelis–Menten parameters (*K*_m_ and *V*_max_) were determined by monitoring the rate of product formation using a Shimadzu UV-2700 spectrometer equipped with a Quantum Northwest TC1 temperature controller to maintain the temperature during measurements. The rate of product formation for the reaction of enzymes with gentisate and salicylate was monitored at 380 or 283 nm (ε_380_^gentisate^ = 2.4 mM^–1^ cm^–1^, ε_283_^salicylate^ = 13.6 mM^–1^ cm^–1^), respectively. Prior to kinetic measurements, buffers were equilibrated with air by mechanical agitation. To maintain the temperature, samples were incubated in a water bath set to the desired temperature. Enzymes were preincubated with the buffer under specific pH and temperature conditions. Following this, a substrate (gentisate or salicylate) was introduced into the cuvette prior to the addition of the preincubated enzyme–buffer mixture, thus initiating the reaction. Each run was repeated 3 times to estimate the experimental error.

### Data Analysis

*K*_m_ and *V*_max_ were determined by fitting the measured initial velocities to the standard Michaelis–Menten equation using Igor Pro 8. Subsequently, *k*_cat_- or *k*_cat_/*K*_m_ - pH profiles were plotted and are fitted to eq 1 to estimate the p*K*_a_ values.^24−26^
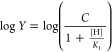
1where *Y* is defined as either *k*_cat_- or *k*_cat_/*K*_m_ and variables [H] and *K*_1_ represent hydrogen ion concentration and the dissociation constant for the observed ionizable group during catalysis, respectively. In the above expression, the variable *C* represents a constant quantity used to scale the maximal kinetic parameter (*k*_cat_- or *k*_cat_/*K*_m_). The standard enthalpy of ionization, Δ*H*_ion_°, was measured by obtaining the p*K*_a_ values at variable temperatures. In accordance with the Van’t Hoff equation, the p*K*_a_ – 1/*T* profile was plotted to extract the thermodynamic parameter by linear regression using eq 2.
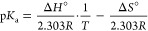
2where *T* is the temperature for the corresponding p*K*_a_ value and *R* is the gas constant.

## Results and Discussion

### Effect of pH on Steady-State Kinetics

**Figure 1:**
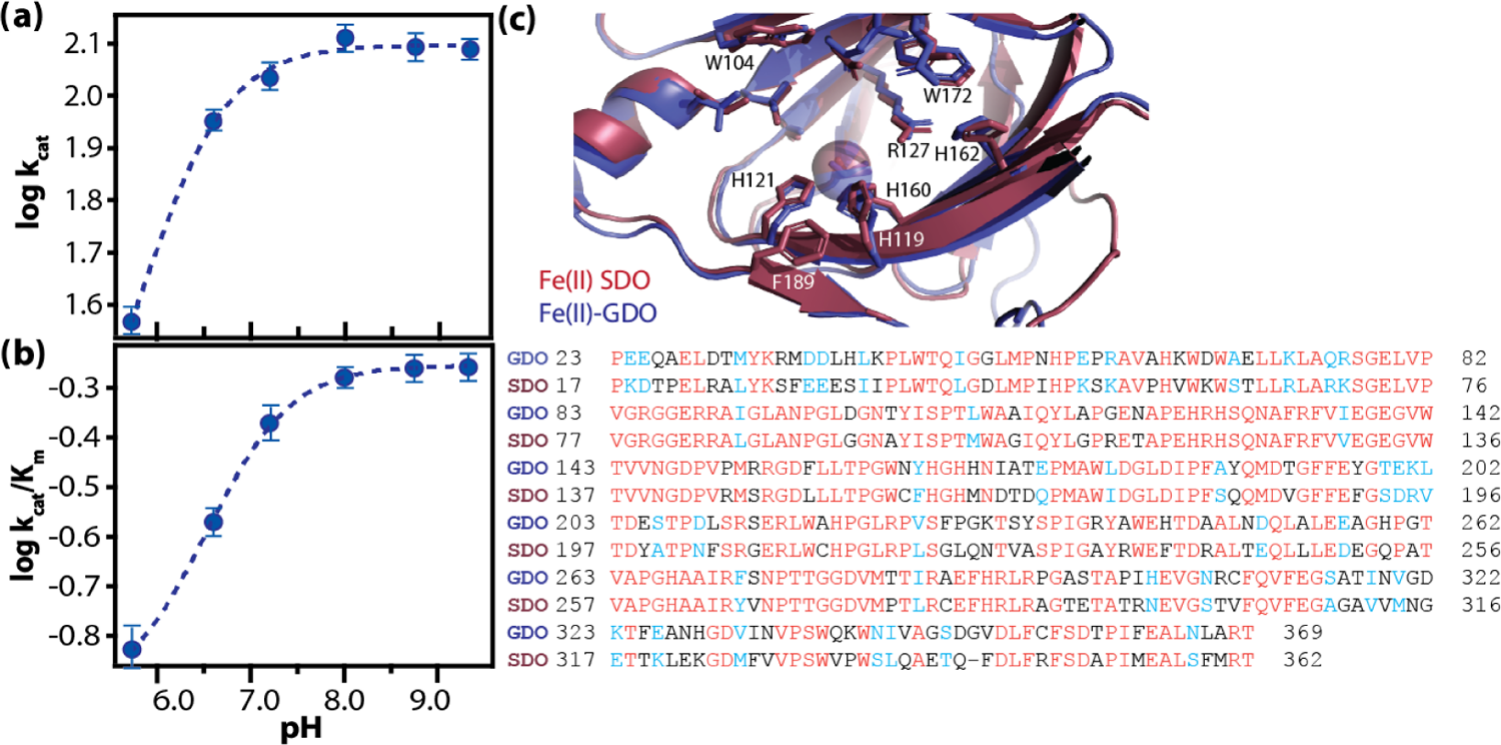
pH dependence of *k*_cat_ (a) and *k*_cat_/*K*_m_ (b) for GDO-catalyzed degradation of gentisate at 25 °C. Each point in the plot was obtained by fitting the standard Michaelis–Menten equation to the initial reaction rate data obtained at a fixed pH value. The uncertainty in the data points was calculated by repeating the Michaelis–Menten experiment at each pH condition in triplicates. The dashed line represents fit to the experimental log(*k*_cat_) and log(*k*_cat_/*K*_m_) vs pH data using eq 1. A summary of the p*K*_a_ values obtained from these fits is presented in Table 1. (c) Top: Overlay of the active sites of GDO (blue; PDB: 3BU7) and SDO (red; PDB: 2PHD); bottom: sequence alignment of the two enzymes (red letters denote identical and blue letters denote similar amino acids).

**Figure 2:**
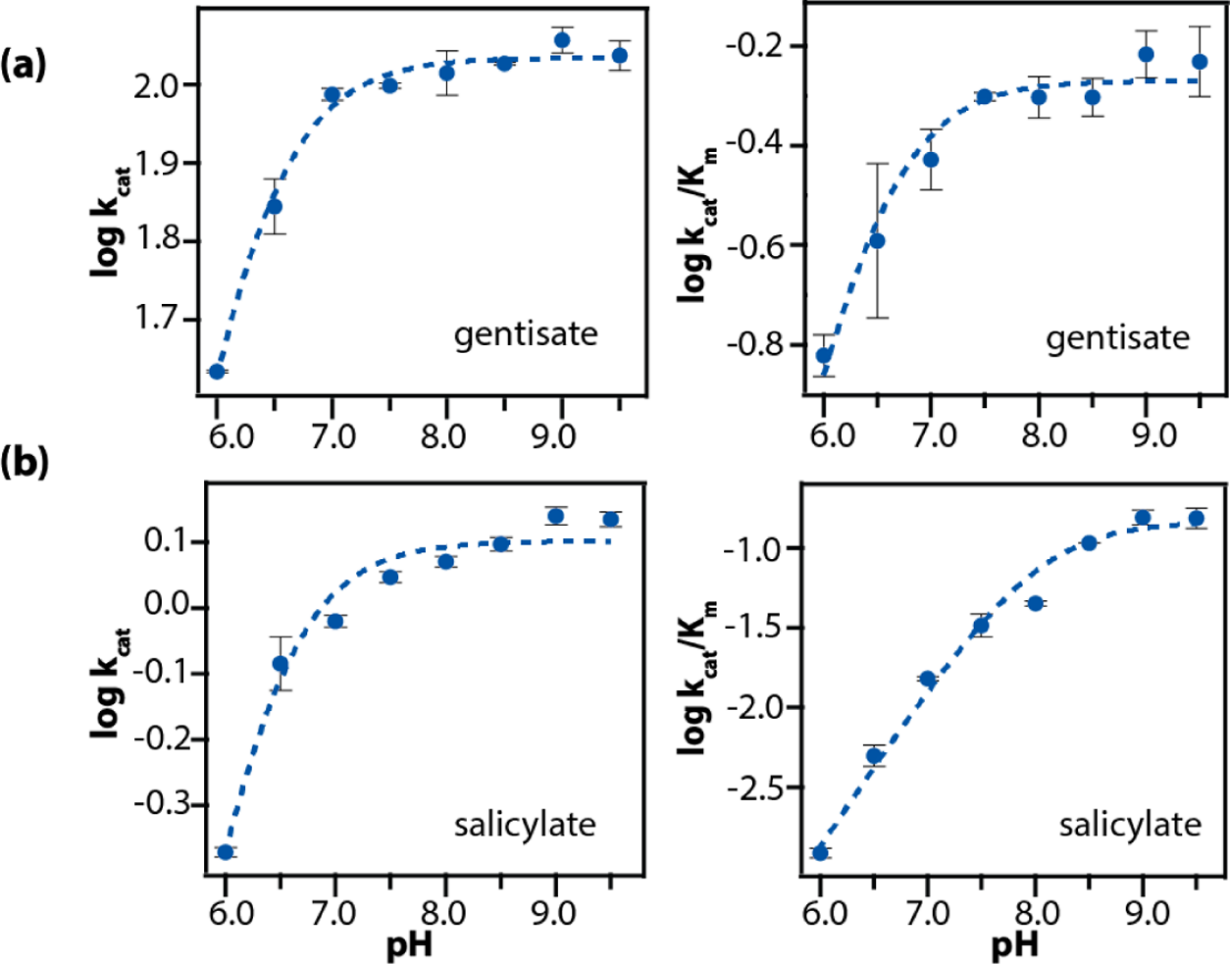
pH dependence of *k*_cat_ and *k*_cat_/*K*_m_ for the SDO-catalyzed degradation of gentisate (a) and salicylate (b) at 25 °C. Each point in the plot was obtained by fitting the standard Michaelis–Menten equation to the initial reaction rate data obtained at a fixed pH value. The uncertainty in the data points was calculated by repeating the Michaelis–Menten experiment at each pH condition in triplicates. The dashed line represents fit to the experimental log(*k*_cat_) and log(*k*_cat_/*K*_m_) vs pH data using eq 1. A summary of the p*K*_a_ values obtained from these fits is presented in Table 1.

The response of variable pH on steady-state turnover by GDO and SDO was evaluated with the dependencies of *k*_cat_ and *k*_cat_/*K*_m_. The initial rates of the reaction of GDO and SDO with gentisate and/or salicylate were measured as a function of pH. The Michaelis–Menten parameters derived from the initial rate of the reaction at a fixed pH value yielded the corresponding points for the plots in Figures 1 and 2. Within the accessible pH range of GDO (5.5–9.5), the value of *k*_cat_ remained constant as the pH was lowered from 9.5 to 7.5; further decrease in the pH showed a decrease in the turnover number (Figure 1a). A similar trend was observed for *k*_cat_/*K*_m_, which remained constant in the pH range of 8.0–9.5 (Figure 1b). Apparent p*K*_a_ values of 6.28 ± 0.10 and 6.86 ± 0.02 were obtained from the plots of log(*k*_cat_) and log(*k*_cat_/*K*_m_) vs pH for the reaction of GDO with gentisate. These results provide evidence of the participation of an ionizable group in the catalysis of gentisate by GDO.

The plots of log(*k*_cat_) and log(*k*_cat_/*K*_m_) vs pH for the reaction of SDO with gentisate exhibited a clear dependence on proton concentration (Figure 2). The overall trend in the pH dependencies of the kinetic parameters was similar to GDO, whereby lowering the pH resulted in attenuated *k*_cat_ and *k*_cat_/*K*_m_ values. Analysis of the log(*k*_cat_) and log(*k*_cat_/*K*_m_) vs pH plot revealed single apparent p*K*_a_ values of 6.16 ± 0.01 and 6.42 ± 0.25, respectively. Given the similarities in the active sites of GDO and SDO (Figure 1c), comparable p*K*_a_ values suggest that identical ionizable groups may be participating in the reaction cycles of the two enzymes. SDO is a unique member of the GDO family owing to its ability to perform C–C bond scission in monohydroxylated substrates such as salicylate. To evaluate the involvement of ionizable protons in SDO-mediated catalysis of monohydroxylated substrates, the response of pH on the steady-state parameters for the reaction of SDO with salicylate was monitored. The pH dependence of the kinetic parameters for the reaction of salicylate with SDO (Figure 2b) exhibited a trend similar to its dehydroxylated counterpart, gentisate. A single proton source event facilitates the degradation of salicylate with an apparent p*K*_a_ of 6.30 ± 0.06 derived from the dependence of log(*k*_cat_) with pH. The fitted parameters for all of the reactions are reported in Table 1. The differences in apparent p*K*_a_ values determined from log(*k*_cat_) and log(*k*_cat_/*K*_m_) plots bear information on the relative rates of the steps involved during catalysis or alternatively may arise due to pH-dependent variation in the environment of the enzyme–substrate complex. The large difference in the apparent p*K*_a_ for the catalysis of salicylate by SDO likely originates from mechanistic differences in the degradation of this substrate compared to gentisate. To summarize, the reactions catalyzed by both GDO and SDO are enabled by a single proton transfer. The p*K*_a_ of the proton source is comparable for the reaction of gentisate by the two enzymes, suggesting their chemical similarity. Similar to gentisate, the SDO-catalyzed oxidation of salicylate also requires a single pH-sensitive step. These results demonstrate that while the reaction mechanism of the SDO-catalyzed degradation of salicylate may differ from that of gentisate or GDO, both reactions require the assistance of a proton source for completion.

**Table 1:** Steady-State Kinetic Parameters for Reactions Catalyzed by GDO and SDO as a Function of pH^a^

enzyme	kinetic constant	gentisate	salicylate
GDO	log(*k*_cat_)		
	p*K*_a_	6.28 ± 0.10	no activity
	log(*k*_ca_/*K*_m_)		
	p*K*_a_	6.86 ± 0.02	no activity
SDO	log(*k*_cat_)		
	p*K*_a_	6.16 ± 0.01	6.42 ± 0.25
	log(*k*_ca_/*K*_m_)		
	p*K*_a_	6.30 ± 0.06	7.89 ± 0.12

a GDO shows no activity toward salicylate, while SDO can oxidize both gentisate and salicylate.

### Identity of the Proton Source in the Reaction Cycle of GDO and SDO

As expected, the active sites of SDOs and GDOs bear several ionizable amino acids. However, the contributions of these ionizable residues toward reaction catalysis are not fully understood. Previous computational studies have suggested that the reaction cycle of SDO may not require transfer of a proton for the ring fission.^19^ Based on these computational studies, ionizable amino acids in the catalytic cavity could instead assist in substrate selectivity and optimal binding for reaction progress. However, in contrast to previous studies, our experimental results show evidence of participation of a proton source. Below, we consider the structural features of the catalytic cavities in SDO and GDO to evaluate the chemical nature of the observed proton source.

While substrate-free and substrate-bound structures of SDO from *P. salicylatoxidans* and its numerous mutants have been extensively characterized by X-ray crystallography,^14,27,28^ limited structural information is available for GDOs. For GDO, crystal structures of substrate-free forms of the enzyme from *E. coli* O157:H7 (*ec*GDO)^29^ and *Silicibacter pomeroyi* (*sp*GDO)^30^ have been reported, but substrate ligated forms have not been structurally characterized. A comparison of the available crystal structures suggests that overall SDO and GDO share similar folds in their cupin domains. Furthermore, amino acid sequence alignments of these enzymes suggest a high degree of sequence homology between SDO extracted from *P. salicylatoxidans* and GDOs originating from other bacteria.^31^ This sequence similarity is particularly relevant for residues that compose the catalytic pockets. For instance, Leu38, Gln108, Ala125, Arg127, His162, Trp172, Asp174, and Leu176 are conserved among all GDOs.^14^ Concomitantly, key variations have also been noted.^27,28^ For instance, in SDO/*ec*GDO/*sp*GDO Met46/Asp45/Val32, Ala85/Val85/Leu71, Trp104/Phe104/Try89, and Phe189/Tyr109/Ala174, modifications are observed.

**Figure 3:**
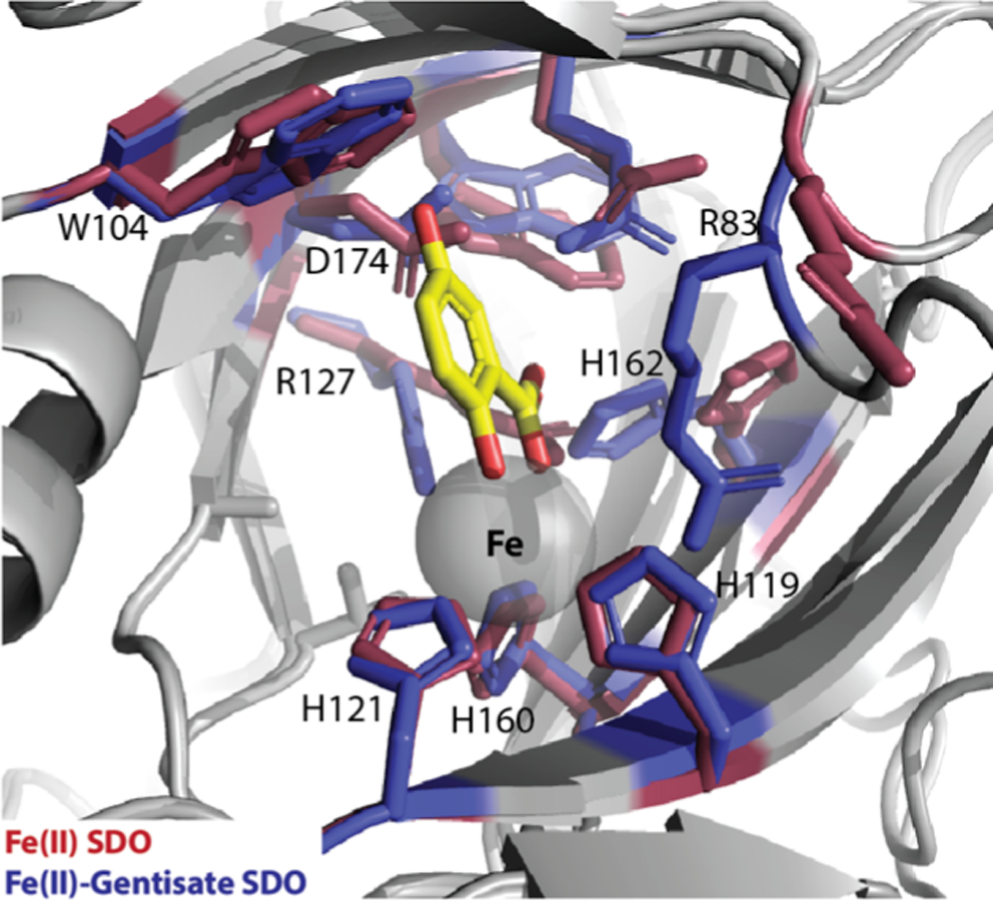
Overlay of the crystal structures of substrate-free (red; PBD: 2PHD) and gentisate-bound (blue; PDB: 3NL1) SDO showing the active site residues and their observed displacement upon substrate binding.

Crystal structures of gentisate, salicylate, and 1-hydroxy-2-naphthoate bound adducts of SDO indicate distinct conformational changes for key active site residues upon substrate binding.^27^ Bidentate ligation of the substrate at the ferrous center leads to pronounced movement in Arg83, bringing it within a hydrogen-bonding distance to one of the carboxyl oxygens of the substrate. Similarly, substrate binding-initiated reorientation of His162 allows for the formation of a hydrogen bond with the other carboxyl oxygens of the substrate and Asp174, Gln108, Trp172, and Arg127 positions to form stable contacts with the substrate (Figure 3). It is noteworthy that all of these residues are strictly conserved among all known GDOs. The following residues with ionizable side chains are present within 5 Å of the substrate and/or ferrous centers in the substrate (gentisate and salicylate)-bound crystal structures of SDO: Arg83, Arg127, His162, and Asp174 in addition to Fe-coordinating His119, His121, and His160.

The data presented herein provides evidence of a proton source with p*K*_a_ ∼ 6.0 in the reactions catalyzed by GDO and SDO (Table 1). This apparent p*K*_a_ value is highly suggestive of the involvement of a histidine residue, thereby implicating His162. In agreement with this proposal, previous docking calculations have suggested that either His162 and/or Arg127 could participate in the catalytic reaction.^16^ In addition to the strong precedence of the role of histidine residues in heme and nonheme iron enzymes, for related extradiol dioxygenases such as HPCD, a histidine residue (His200) has been shown to play a critical role as a proton mediator by stabilizing the catalytic intermediates. Interestingly, an overlay of the crystal structures of substrate-bound SDO and HPCD suggests that His162 (in SDO) and His200 (in HPCD) are oriented similarly in the active site cavity. Eppinger et al. analyzed His162Ala and His162Phe variants of the enzyme; however, these studies were unable to conclusively establish its role in assisting catalysis.^32^ Potential contributions of Arg residues (Arg83 and Arg127) could not be evaluated by employing site-directed mutagenesis as altering these amino acids resulted in a complete loss of enzymatic activity.^32^

**Figure 4:**
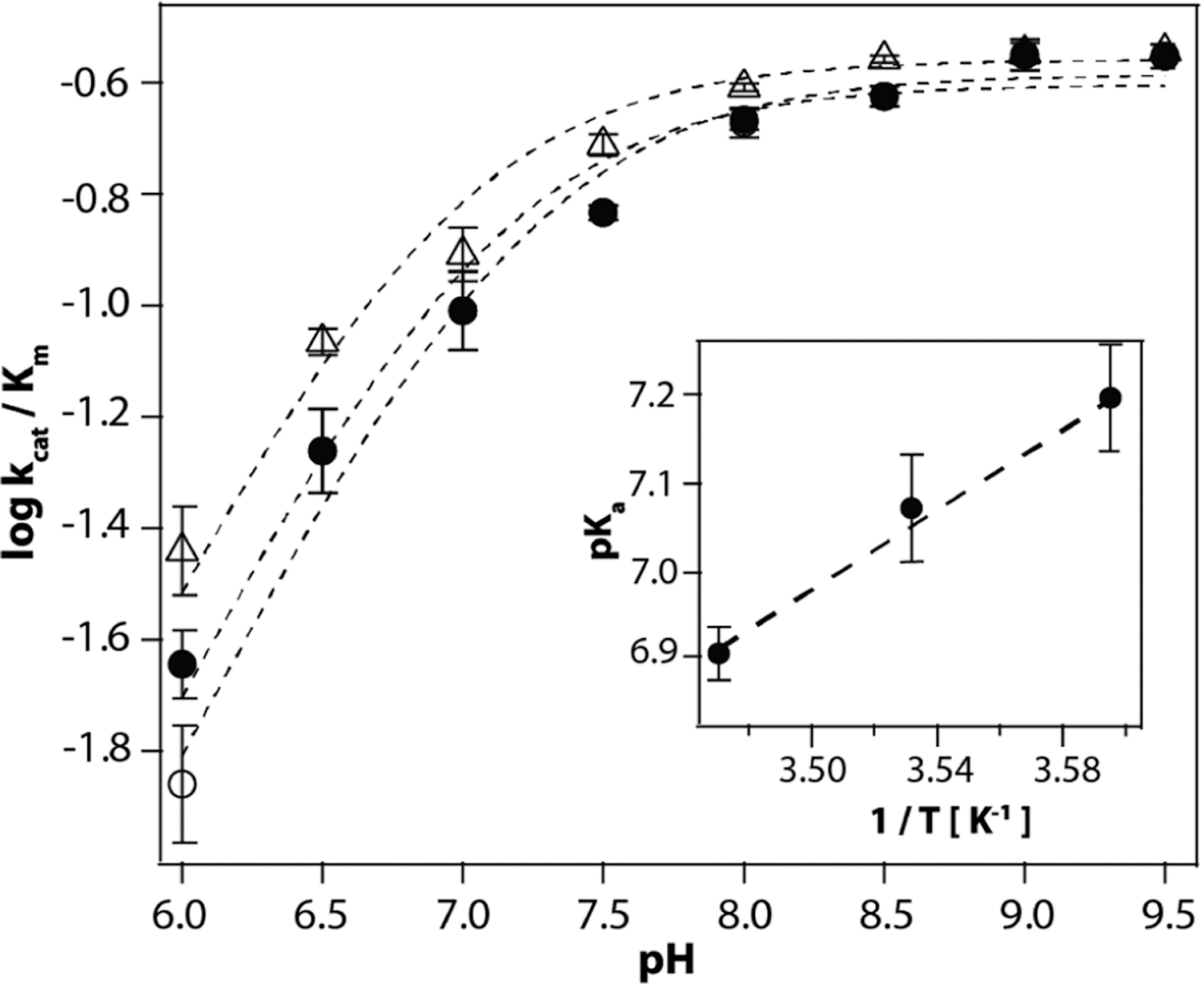
pH dependence of *k*_cat_/*K*_m_ for the SDO-catalyzed degradation of gentisate at 5 °C (open circles), 10 °C (solid circles), and 15 °C (open triangles). Each point in the plot was obtained by fitting the standard Michaelis–Menten equation to the initial reaction rate data obtained at a fixed pH value and temperature. The uncertainty in the data points was calculated by repeating the Michaelis–Menten experiment at each pH condition in duplicates or triplicates. The dashed line represents fit to the experimental data using eq 1. The inset shows the plot of calculated p*K*_a_ values versus 1/*T*, with the dashed line representing the best fit for a linear dependence.

Owing to the high susceptibility of p*K*_a_ in the local environment of an ionizable group, wide variation in apparent p*K*_a_ values can be observed for a given amino acid in biological molecules. Therefore, p*K*_a_ values alone cannot be used to determine the identity of the proton shuffler. In contrast, enthalpy of ionization (Δ*H*_ion_°) values remain relatively invariant to the electronic environment with common biological moieties (amino acid side chains) exhibiting distinct Δ*H*_ion_° values (Table S1).^33^ We determined Δ*H*_ion_° for the observed proton source in the reaction of SDO with gentisate. Michaelis–Menten parameters were determined from initial substrate consumption rates at fixed pH conditions and at 5, 10, and 15 °C, yielding the corresponding data points shown in Figure 4. These log(*k*_cat_/*K*_m_) vs pH plots at each temperature were fitted to eq 1 to obtain the apparent p*K*_a_ values, which decreased with the increase in temperature (Table S2). The inverse proportionality to the temperature of experimental p*K*_a_ values (Figure 4, inset) is in accordance with the Van’t Hoff equation (eq 2). Additionally, the slope of the plot of p*K*_a_ versus 1/*T* yielded a Δ*H*_ion_° of 51.7 ± 9 kJ/mol.

The pH and temperature dependences of Michaelis–Menten parameters for the reaction of SDO with gentisate show the involvement of an ionizable group with p*K*_a_ ∼ 6.0 and Δ*H*_ion_° = 51.7 kJ/mol. The energy differences between the reactants and the products of the ionization reaction in the absence of a solvent are the primary contributors of ionization enthalpies. In addition to this, extrinsic factors such as environment and solvent effects may also contribute, leading to variations in enthalpic values.^34^ Due to these variations, an unambiguous distinction between ionizable amino acids exhibiting similar Δ*H*_ion_° values (such as histidine, cysteine, tyrosine) cannot be made. However, the significantly higher ionization enthalpy of arginine (Table S1) makes it distinct from other remaining amino acids, allowing for its assignment. While the p*K*_a_ value may implicate a histidine residue, the measured Δ*H*_ion_° is significantly larger than the expected enthalpy of ionization of the imidazolium group of histidine (28–31 kJ/mol).^33^ Furthermore, the experimental Δ*H*_ion_° value is indicative of a guanidinium ion in the arginine residue (50–54 kJ/mol). Given that the intrinsic p*K*_a_ of the arginine side chain in solution ranges between 11.6 and 12.6, our results suggest a large suppression of the apparent p*K*_a_ due to the protein environment in SDO. In light of these results and based on the crystal structure, either Arg83 or Arg127 is the plausible proton mediator in the catalytic cycle of SDO. In previous QM/MM calculations by Roy and Kästner, the catalytic mechanism of SDO was investigated on the computationally modeled SDO oxy adduct.^19^ In this geometry-optimized oxy adduct, Arg127 is present in close proximity to the bound dioxygen and forms a hydrogen bond with the carboxyl oxygen of the substrate. This suggests that Arg127, with its optimal placement to assist in proton transfer and/or stabilize a catalytic intermediate during the reaction, may be responsible for the observed pH dependence in our results instead of Arg83. Lastly, while the strict conservancy of His162 and Arg127 make them potential candidates for the proton source in the reaction of gentisate with GDO (Figure 1), considering the structural similarities between GDO and SDO, it is likely that Arg127 is the proton shuffler in the GDO catalytic cycle. Given the expanded substrate specificity of SDO owing to its ability to degrade monohydroxylated substrates, it has been proposed that the catalytic mechanism of SDO-mediated oxidation of salicylate is distinct from that of gentisate. The studies reported herein suggest that while the intermediatory steps in the catalytic cycles may differ, degradation of both substrates by SDO and gentisate by GDO requires a single proton source.

### Putative Origin of Depressed p*K*_a_ of the Proton Source

With arginine as the likely proton mediator in SDO-mediated ring fission of gentisate, a pertinent question arises: *how and why does nature utilize an arginine residue with a highly suppressed pK*_*a*_*when histidine residues have been implicated for similar proton shuffling in related dioxygenases?* We speculated that the origin and the need for utilizing arginine may arise due to the atypical nature of the primary coordination sphere tethering the Fe cofactor in GDO and SDO. The 2-His-1-carboxylate facial triad is the most abundant scaffold supporting the metal cofactor in nonheme enzymes. In contrast to this ubiquitous motif, several atypically coordinated metal cofactors have also been identified such as 3-His, 3-His-1-carboxylate, and 4-His.^8^ A fundamental difference arising due to these variations in the primary coordination sphere is the charge of the protein-derived metal–ligand complex. For instance, the net charge of the ferrous cluster supported by the 3-His motif (such as in SDO) is +1 more than that of the 2-His-1-carboxylate motif (for example, in HPCD).

In folded proteins, charge–charge interactions can have dramatic effects on the p*K*_a_ values of ionizable groups. Typically, the presence of a positively charged environment diminishes the apparent p*K*_a_, while negatively charged groups elevate the p*K*_a_.^35^ For instance, while the p*K*_a_ of free Glu in solution is 4.07, the lowest observed p*K*_a_ of Glu in the folded protein environment is 2.1 in protein barnase. This depressed p*K*_a_ is supported by positive charges from a nearby lysine and two arginine residues.^36^ Therefore, it is conceivable that the positive charge afforded by the 3-His ferrous coordinating motif helps lower the p*K*_a_ of arginine in SDO and GDO active site cavities compared to free arginine in solution. To test this hypothesis, we generated a 2-His-1-carboxylate variant of GDO in which a metal ligating His119 (SDO numbering) residue was mutated to Asp (herein referred to as H119D-GDO). Similar variants of SDO did not show any appreciable catalytic activity. The Alphafold^37^-predicted structure of this variant showed no major conformational change due to this mutation (Figure S1).

**Figure 5:**
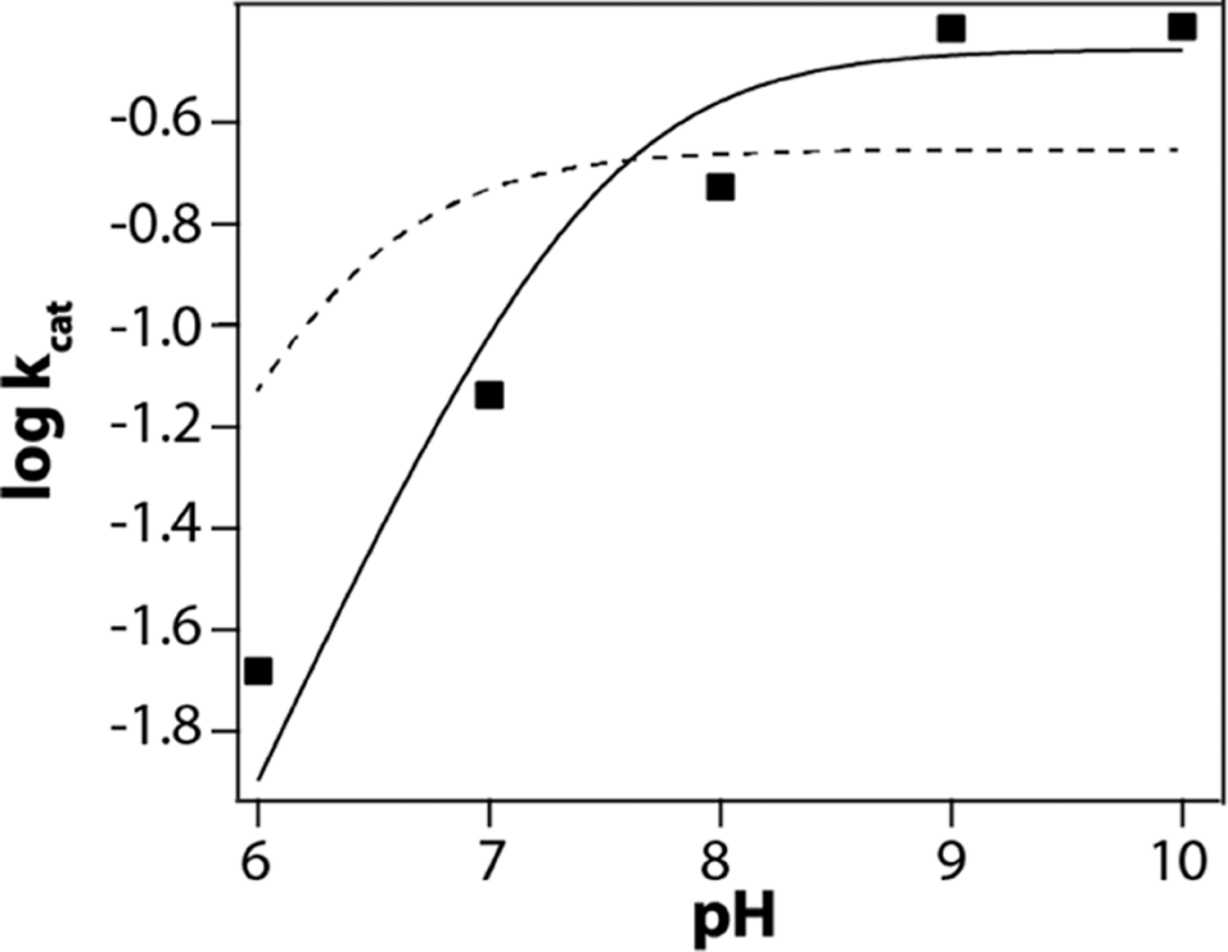
pH dependence of *k*_cat_ for the H119D-GDO-catalyzed degradation of gentisate at 25 °C. Each point in the plot was obtained by fitting the standard Michaelis–Menten equation to the initial reaction rate data obtained at a fixed pH value. The solid line represents the best fit to the experimental data using eq 1. The dashed line represents a fit to eq 1 with p*K*_a_ = 6.3 to demonstrate that the observed p*K*_a_ is greater than ∼6.0.

To determine the p*K*_a_ of the ionizable group in the catalysis by H119D-GDO, similar to the wildtype protein, the response of catalytic turnover on pH was monitored. Figure 5 shows the plot of log(*k*_cat_) vs pH for this variant. As in the wildtype protein, lowering the pH resulted in reduced *k*_cat_ values, with clear evidence of one proton transfer during the reaction. The experimental data can be best recapitulated with a p*K*_a_ of 7.30 ± 0.21. This p*K*_a_ value is clearly higher than that observed for the wildtype protein (p*K*_a_^GDO^ = 6.28). Interestingly, a p*K*_a_ of ca. 7.65 has also been reported for the proton mediator in the related 2-His-1-carboxylate dioxygenase, HPCD.^38^ These previous studies on HPCD and current results on the 2-His-1-carboxylate variant of GDO provide support to our hypothesis that the atypical 3-His motif of GDO and SDO may play a critical role in suppressing the p*K*_a_ of the proton mediator required in the reaction catalysis. Furthermore, this could also explain why GDO recruits arginine instead of the customary histidine residue as the proton source: given that the 3-His motif is poised to suppress the p*K*_a_ of the proton shuffler in the reaction, a histidine residue will exhibit a depressed p*K*_a_, which may not be optimal for the reaction. The inherently higher p*K*_a_ of an arginine residue may negate the influence of the positively charged environment afforded by the 3-His motif, allowing it to bear optimal p*K*_a_ for catalysis. Lastly, we note that the protonation of the bound substrate molecule will also affect the overall charge of the metal–ligand cluster. While the protonation states of bound substrate molecules are difficult to determine, previous studies on HPCD and SDO have shown that in both enzymes, the charge of the bound nitrosubstituted substrate analogue is the same.^21,39^ Future studies investigating the protonation states of native substrates in enzymes with variable coordination spheres could provide greater clarity on this issue. Taken together, our results offer insights into the phylogenetic relevance of conserved arginine residues in GDOs.

### Implications for the Catalytic Mechanism of GDO/SDO

The reaction catalyzed by GDOs is distinct from related dioxygenases such as HPCD. In GDO (and SDO), the Fe cofactor assists in the aromatic bond scission between positions 1 and 2 of the aromatic ring, in contrast to the bond cleavage between positions 2 and 3 of the catechol-derived substrate by HPCD. While several studies in the literature have focused on the reaction mechanism of extradiol ring cleavage by HPCD, no experimental mechanistic studies have been performed on GDO and SDO. Both experimental and computational studies exploring the catalytic mechanism of HPCD have established clear evidence of the role of an acid–base catalyst in the reaction.^12,38,40^ A histidine residue placed in the proximity of the bound dioxygen moiety in HPCD stabilizes the superoxo and hydroperoxo intermediates in the catalytic cycle, thereby playing a critical role in substrate and oxygen activation. In addition to HPCD, histidine residues have been implicated as acid–base catalysts in the reaction cycles of several other metalloenzymes.

For GDO and SDO, in the absence of experimental reports, several computational endeavors have attempted to evaluate the reaction mechanism of SDO primarily due to the availability of enzyme–substrate bound crystal structures of the enzyme. However, these studies have yielded contradictory results. QM/MM simulations by Kästner and Roy suggested that the catalysis carried out by SDO does not require a proton source, suggesting that the reaction mechanism undergoes a pathway different from that of HPCD by utilizing a strong covalent interaction between the ferrous center and the bound dioxygen moiety.^19^ A subsequent QM/MM study by Ryde and Dong proposed a contradictory mechanism, in which His162 was suggested to assist in oxygen activation by participating as an acid–base catalyst during the reaction.^20^ In this study, calculations were performed on various protonated states of His162 to evaluate its role in catalysis, and Arg127 was proposed to assist by hydrogen-bond formation with dioxygen.

**Figure 6:**
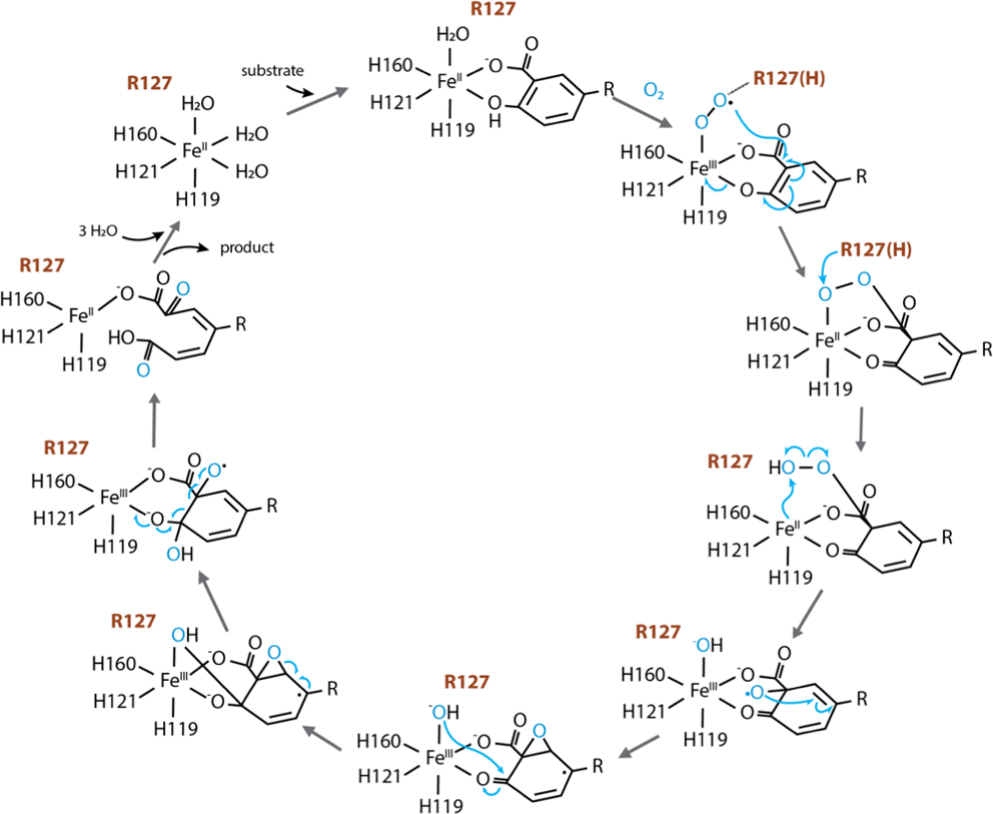
Proposed reaction mechanism for the catalysis of gentisate and/or salicylate by GDO (and SDO).

The pH dependence of the Michaelis–Menten parameters in our experiments demonstrates that a proton shuffler is indeed needed to assist in the reaction catalysis. Furthermore, the temperature and pH dependence of the Michaelis–Menten parameters show that the proton source is not a histidine residue, making Arg127 the most likely candidate based on its proximity to the dioxygen moiety. With these results, a reaction mechanism of SDO-assisted ring fission considering the role of a proton shuffler can be proposed, based on the crystallographically observed reaction intermediates and computationally postulated reaction mechanism of the homogentisate 1,2-dioxygenase.^41,42^ In this proposal, the oxy adduct formed upon dioxygen binding to the enzyme–substrate complex may be stabilized by the arginine (Arg127) residue. Subsequent cleavage of the C–C double bond between positions 1 and 2 may produce the hydroperoxo complex upon a proton donation by arginine. Subsequent homolytic cleavage of the O–O bond will lead to the final product. Future mechanistic and spectroscopic studies will be needed to provide further support for this mechanism (Figure 6).

## Conclusions

GDO (and SDO) bear an atypical primary coordination sphere and catalyze the bond cleavage between positions 1 and 2 of their aromatic substrate, making them distinct from other related extradiol dioxygenases. In this report, we provide evidence of proton-mediated reaction catalysis by this class of enzymes. Our results clarify the discrepancies in the literature arising from computational studies. pH dependence of steady-state parameters shows that a single proton transfer event takes place during GDO- and SDO-mediated O_2_ activation and oxidation of gentisate. Similarly, studies show that a proton source is also needed for the SDO-mediated catalysis of salicylate. Analysis shows that the proton source bears a p*K*_a_ of 6–7 in these reactions, making the histidine residue a likely candidate owing to its vicinity in the enzymatic cavity. However, as opposed to the customary histidine residue typically implicated in the acid–base catalysis of related dioxygenases, temperature and pH dependence of Michaelis–Menten parameters suggest that the observed proton source is arginine. We suggest that the atypical 3-His coordination sphere in GDO and SDO, which bears an overall net positive charge for the metal–ligand cluster, may contribute toward the suppression of arginine p*K*_a_. pH dependence of the 2-His-1-carboxylate variant of GDO provides support to this argument and offers insights into the phylogenetic relevance of conserved arginine residues in GDOs. Based on these results, a reaction mechanism considering the role of the observed proton source is proposed.

## Supporting Information Available

Ionization enthalpies for typical ionizable groups in enzyme-active sites; temperature dependence of p*K*_a_ values for the reaction of SDO with gentisate; and overlay of the GDO crystal structure with the Alphafold-predicted structure of H119D-GDO (PDF)
