# Supplementary material for: Proton Transfer via Arginine with Suppressed pKa Mediates Catalysis by Gentisate and Salicylate Dioxygenase
Source: J Phys Chem B. 2024 Jul 9;128(28):6797–805. doi: 10.1021/acs.jpcb.4c03164 (PMC11264262; doi:10.1021/acs.jpcb.4c03164)
Supplement: Supplementary file 1 — jp4c03164_si_001.pdf [file jp4c03164_si_001.pdf]

Supporting Information for

Proton Transfer via Arginine with Suppressed pK_a Mediates Catalysis by Gentisate and Salicylate Dioxygenase

Qian Wang[‡], Kamal Rai[‡], Aleksey Aleshintsev[‡,⊥], Eric Jin[§] and Rupal Gupta[‡,⊥,*]

[‡]Department of Chemistry, College of Staten Island, City University of New York, New York, 10314, United States

[§]Staten Island Technical High School, Staten Island, NY 10306, United States

[⊥]Ph.D. Programs in Biochemistry and Chemistry, The Graduate Center of the City University of New York, United States

***Corresponding author**: Rupal Gupta, Department of Chemistry, College of Staten Island, The City University of New York, USA

Table S1. Ionizable groups typically present in enzymatic active sites.[1]

Group	pK_a	ΔH^0_{ion}(kJ/mol)
α-Carboxyl (at end of polypeptide chain)	3.0–3.2	±6
β- or γ-Carboxyl (of aspartic or glutamic acid)	3.0–5.0	±6
Imidazolium (of histidine)	5.5–7.0	28–31
α-Amino (at end of polypeptide chain)	7.5–8.5	42–54
ε-Amino (of lysine)	9.5–10.6	42–54
Sulfhydryl (of cysteine)	8.0–8.5	27–29
Phenolic OH (of tyrosine)	9.8–10.5	25
Guanidinium (of arginine)	11.6–12.6	50–54

Table S2. Temperature dependence of observed pK_a values for the reaction of SDO with gentisate.

Temperature (K)	pK_a
278	7.2 ± 0.06
283	7.06 ± 0.05
288	6.87 ± 0.03

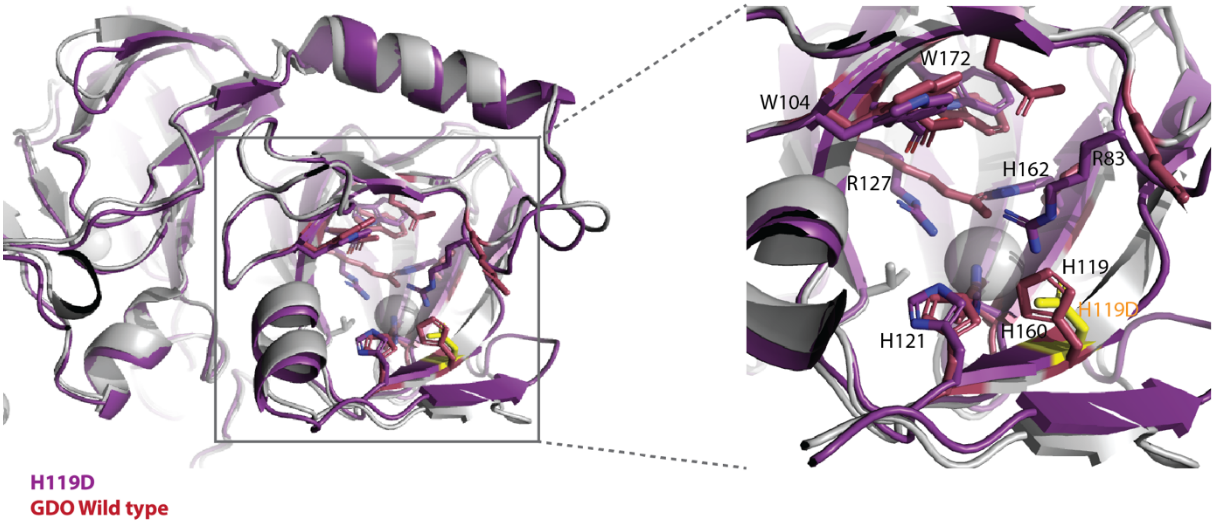

Figure S1. Overlay of GDO crystal structure (red; PDB: 3BU7) and Alphafold predicted structure of H119D-GDO.

References:
(1) Segel, I. H. *Enzyme Kinetics: Behavior and Analysis of Rapid Equilibrium and Steady-State Enzyme Systems*; Wiley, 1993.
